# Serum iron and risk of nonalcoholic fatty liver disease and advanced hepatic fibrosis in US adults

**DOI:** 10.1038/s41598-021-89991-x

**Published:** 2021-05-17

**Authors:** Huan-Huan Yang, Guo-Chong Chen, De-Ming Li, Lei Lan, Li-Hua Chen, Jia-Ying Xu, Li-Qiang Qin

**Affiliations:** 1 Department of Nutrition and Food Hygiene, School of Public Health, Soochow University, 199 Ren’ai Road, Suzhou, 215123 China; 2 Department of Epidemiology and Population Health, Albert Einstein College of Medicine, New York, USA; 3 State Key Laboratory for Diagnosis and Treatment of Infectious Diseases, The First Affiliated Hospital, College of Medicine, Zhejiang University, Hangzhou, China; 4 Department of Nutrition and Food Hygiene, School of Public Health, Nantong University, Nantong, China; 5 State Key Laboratory of Radiation Medicine and Protection, School of Radiation Medicine and Protection, Soochow University, Suzhou, China

**Keywords:** Liver diseases, Risk factors

## Abstract

Epidemiological evidence on the relationship between serum iron and liver diseases is limited. This study aims to investigate whether serum iron is associated with nonalcoholic fatty liver disease (NAFLD) and advanced hepatic fibrosis (AHF). Cross-sectional data for adults aged ≥ 18 years who participated in the National Health and Nutrition Examination Survey (NHANES) from 1999 to 2018 were analyzed. Odds ratio (ORs) and 95% confidence intervals (CIs) of NAFLD and AHF associated with serum iron were estimated using multivariable logistic regression models. A total of 18,031 males and 18,989 females were included in the analysis. After multivariable adjustment for potential confounders, serum iron was significantly and inversely associated with NAFLD in both genders (*P-trend* < 0.001) and AHF in females (*P-trend* = 0.018). Compared to the bottom quartile, those in higher quartiles of serum iron had no significant ORs for AHF in males, but the trend across the quartiles was significant (*P-trend* = 0.046). In conclusion, higher serum iron level was associated with lower risk of NAFLD in males and females, and with lower risk of AHF in females but not in males. No significant racial/ethnical differences in these associations were observed.

## Introduction

Iron is an essential nutrient integral to various metabolic processes^1^. Serum iron (i.e., transferrin bound iron) can be delivered to cells or organ where needed, and can recycle back to the blood, and thus acts as a clearing house in regulating iron distribution in the body^1^. However, labile plasma iron has a high propensity for redox activities, and excessive iron may lead to highly reactive chemical entities through Fenton reaction in cells. The liver is a vital metabolic organ that plays a major role in maintaining iron metabolism^2^.

Accumulating evidence indicates that excess iron deposition in the liver induces liver toxicity^3,4^. Thus, maintenance of body iron homeostasis is crucial for liver health. Especially in patients with chronical liver diseases, iron status should be anticipated and closely monitored to prevent them from progression to cirrhosis or hepatocellular carcinoma (HCC). However, the invasive liver biopsy required for liver iron testing is always impracticable for the general population or patients with chronical liver diseases, such as nonalcoholic fatty liver disease (NAFLD).

Biochemical measures of iron status, such as iron, ferritin, and transferrin receptor (TFR) are the most useful clinical markers for iron homeostasis, easily obtained and minimally invasive^5^. One research conducted by Ribot-Hernández *et.al* showed an inverse association between serum iron and chronic alcoholic liver disease^6^. However, there were also inconsistent conclusion, such as Bertol *et.al* found that serum iron was not correlated with biopsy-proven NAFLD staging^7^; serum iron has also been reported to be positively associated with alanine aminotransferase (ALT) concentration based on the third U.S. National Health and Nutrition Examination Survey (NHANES III)^8^. As far as we know, there is no epidemiological evidence available about the association between serum iron and NAFLD or advanced hepatic fibrosis (AHF).

There were also studies indicated that serum ferritin was positively associated with risk of NAFLD^9–11^ and liver fat content^12^. However, a study of 30 patients with biopsy-proven NAFLD/non-alcoholic steatohepatitis (NASH) showed that there is no significant correlation between serum ferritin and the stage of fibrosis^13^. Taken together, the relationship between serum iron status and risk of NAFLD or AHF remains limited and inconclusive. Moreover, gender difference was observed in the control of iron metabolism due to iron losses during menstruation in females and the distinct sex hormone between males and females. Evidence demonstrated that females are particularly vulnerable to iron deficiency^14^.

Therefore, we aim to assess the cross-sectional relationships between serum iron status and risk of NAFLD and AHF, and the difference between males and females using data from the NHANES, a well-designed population-based study including nationally representative US adults.

## Methods

### Subjects

NHANES is a complex, stratified, multistage, and probability-cluster designed program of the National Center for Health Statistics, which aimed to assess the health and nutritional status of adults and children in the US^15^. The surveys were approved by the National Center for Health Statistics’ Ethics Review Board. All methods were performed in accordance with the relevant guidelines and regulations. Informed consent was obtained from all participants.

Data from ten cycles of the NHANES that were collected during 1999–2018 were included in this analysis. The data of serum iron is available for all the ten cycles, while ferritin is available for the years 1999–2010 and 2015–2018, and TFR for the years 2003–2010 and 2015–2018. Included participants were adults, who were 18 years or older and completed at least one 24-h dietary recall. Participants were excluded if they: (1) had elevated alcohol intake (> 21 standard drinks per week in males; or > 14 standard drinks per week in females)^16^; (2) missed information required for the definitions of NAFLD or AHF (described below); (3) had positive hepatitis B surface antigen, or hepatitis C virus RNA; (4) were pregnant women; (5) had self-reported cancer; (6) had unreliable dietary recall status, or abnormal energy intake (< 800 kcal/day or > 4200 kcal/day); (7) taken prescribed medicines in one month that can affect hepatic steatosis^16^ (see Supplementary Fig. S1 online).

### Serum iron status and other laboratory measurements

Following the primary screening questionnaire, all participants who satisfied the inclusion criteria were invited to the mobile examination center (MEC) for further measurement. Physiological measurements, specimen collection, and laboratory tests were performed by professional medical staff according to MEC laboratory procedures manual. Serum iron, ferritin, TFR, ALT, aspartate aminotransferase (AST) and γ-glutamyl transferase (GGT) were measured using the refrigerated serum samples. Serum iron was measured by collaborative laboratory services using timed-endpoint method. ALT, AST, and GGT were measured using Beckman UniCel DxC800 Synchron by the same laboratory. Ferritin was measured using Roche Elecsys-170, and the method for TFR is immuno-turbidimetry using Roche kits on the Hitachi 912 chemistry analyzer (2003–2008) or Hitachi Mod P clinical analyzer (2009–2010) or Cobas C501 clinical analyzer (2015–2018). Whole blood lead (Pb) and cadmium (Cd) content were measured using mass spectrometry. Detailed instructions on sample collection and other methodological approaches can be found at https://wwwn.cdc.gov/nchs/nhanes/AnalyticGuidelines.aspx.

### Measurements of other covariates

Sociodemographic characteristics and lifestyle were collected using a standardized self-administered questionnaire. Weight, height, and waist circumference (WC) were measured by well-trained health technologists according to anthropometry procedure manual. Body mass index (BMI) was calculated as weight (kg) divided by height squared (m^2^). Leisure time activity levels were measured by physical activity questionnaire, which is based on the Global Physical Activity Questionnaire.

Information on dietary intake was estimated from What We Eat in America linked to Food and Nutrient Database for Dietary Studies, which consists of two 24-h dietary recalls conducted by trained interviewers and nutrient values for foods and beverages reported in What We Eat in America. The healthy eating index (HEI) was calculated on the basis of 37 United States Department of Agriculture food-pattern components from the Food Patterns Equivalents Database^17^ (see Supplementary Table S1 online).

### Outcome definitions

Noninvasive diagnostic indexes, such as the fatty liver index (FLI) and NAFLD fibrosis score (NFS) have been extensively used for liver disease detection. We used FLI, a validated diagnostic index, to define NAFLD^18^. FLI score of ≥ 60 was assumed to have NAFLD. NFS was chosen for defining AHF in this study due to it showed higher predictive performance in NAFLD population^16,19,20^. Participants have AHF if they met NFS > 0.676 in the presence of NAFLD. Formulas of FLI and NFS are as follows^18,19^:

$$ {\text{FLI}} = ({\text{e}}^{{0.953 \times {\text{loge }}\left( {{\text{TG}}} \right) + 0.139 \times {\text{BMI}} + 0.718 \times {\text{loge }}\left( {{\text{GGT}}} \right) + 0.053 \times {\text{WC}}{-}15.745}} )/({1} + {\text{e}}^{{0.{953} \times {\text{loge }}\left( {{\text{TG}}} \right) + 0.{139} \times {\text{BMI}} + 0.{718} \times {\text{loge }}\left( {{\text{GGT}}} \right) + 0.0{53} \times {\text{WC}}{-}{15}.{745}}} ) \times {1}00; $$

$$ \begin{gathered} {\text{NFS}} = - {1}.{675} + 0.0{37} \times {\text{age}} + 0.0{94} \times {\text{BMI}} + {1}.{13} \hfill \\ \times {\text{impaired fasting glycemia or diabetes }}\left( {{\text{yes}} = {1},{\text{ no}} = 0} \right) \hfill \\ + 0.{99} \times {\text{AST}}/{\text{ALT ratio}} - 0.0{13} \times {\text{platelet}} - 0.{66} \times {\text{albumin}}. \hfill \\ \end{gathered} $$

Here, diabetes was defined as glycated hemoglobin level ≥ 6.5%, or current use of antidiabetic medication, or self-reported diagnosed diabetes^21^.

### Statistical analysis

All analyses accounted for the complex survey design of the NHANES and weighting variables. We chose the MEC subsample weights (WTMEC4YR for 1999–2002 and WTMEC2YR for 2003–2018) in this analysis because serum biomarkers were used as major indicators in this study. These sampling weights were recalculated to account for combining 10 NHANES cycles for serum iron, 8 cycles for serum ferritin, and 6 cycles for TFR sampling strategy.

Sex-specific quartiles for serum iron were created and all data were analyzed separately in males and females because serum iron level was much higher in males than in females. Differences for categorical variables across quartiles of serum iron were compared using χ2 tests and presented as numbers (n) and percentage (%). Quantitative variables were compared using linear regression models, and data were expressed as mean ± standard error. To test the relationship between serum iron and risk of NAFLD and AHF, we applied multivariable logistic regression models. Potential confounding factors were gradually included in three models with increasing degrees of adjustment. Model 1 was adjusted for age (years), race/ethnicity (Mexican American, other Hispanic, non-Hispanic white, non-Hispanic black, other race), marital status (married or cohabitation, widowed or separated, never married), education level (less than 12th grade, high school or equivalent, college graduate or above), and family poverty income ratio (PIR). Model 2 was further adjusted for smoking status (never, ever, current), alcohol intake (drinks/week), leisure-time physical activity (MET-min/day), total energy intake (kcal/day), and HEI-2015 score. In addition, metals Pb (μg/dL) and Cd (nmol/L), which have shown to have significant hepatic toxicity^22^, were included in the model 3. Then, the continuous variable of the serum iron, ferritin and TFR were log-transformed prior to further analysis. Odds ratios (OR) and 95% confidence interval (CI) of NAFLD and AHF associated with per standard deviation (SD) increment (log-transformed data) of serum iron, ferritin and TFR were reported. Further, serum iron (original data) was divided into quartiles and the ORs with 95% CI were calculated across the quartiles.

The associations between serum iron (per SD increment) and risk of NAFLD and AHF was further assessed in subgroup of population with different racial/ethnical backgrounds, and potential interactions were tested. Analyses were conducted using SAS version 9.4 (SAS Institute Inc., Cary, North Carolina), and *P* value < 0.05 (two-tailed) was considered as statistically significant. Figures were drawn in RStudio (Version 1.3.1056) with the forestplot packages.

## Results

We identified 37,020 eligible participants, including 18,031 males and 18,989 females. These participants represented a weighted population of 74,133,647 and 79,945,020 noninstitutionalized US males and females respectively. Mean serum iron concentrations were higher in males (95.32 ± 0.41 μg/dL) than in females (80.45 ± 0.38 μg/dL). Prevalence of NAFLD was 50.4% in males and 36.6% in females, and that of AHF was 2.6% and 2.5% respectively. Tables 1 and 2 present participant characteristics according to sex-specific quartiles of serum iron. More than 60% of the population was non-Hispanic white, and the rate increased with ascending serum iron quartiles. With higher levels of serum iron, males were more likely to be never married; however, females were more likely to be married or in a state of cohabitation. Compared with quartile 1, higher serum iron was associated with higher levels of education, family PIR, HEI-2015 score, leisure-time physical activity, lower energy intake, and BMI in both genders. Serum iron also showed a significantly positive correlation with ALT, AST, GGT, Pb in males and AST, GGT, Pb in females. Without adjustment for confounders, serum iron was negatively associated with the risk of NAFLD and AHF.

**Table 1:** Basic characteristics of participants according to quartiles of serum iron in males.

	Quartile 1	Quartile 2	Quartile 3	Quartile 4	P*
Serum iron (μg/dL)	< 70.5	70.5–90.3	90.4–113.9	≥ 114.0	
Age (year)	44.42 ± 0.28	44.72 ± 0.31	44.58 ± 0.32	41.84 ± 0.36	< 0.001
**Race (n, %)**					
Mexican American	793 (8.4)	885 (9.3)	841 (9.2)	1117 (11.5)	< 0.001
Other Hispanic	399 (6.2)	356 (5.7)	345 (5.9)	348 (5.8)	
Non-Hispanic White	1860 (63.8)	1904 (67.9)	1916 (69.5)	1946 (70.1)	
Non-Hispanic Black	1363 (14.6)	988 (10.7)	732 (8.0)	544 (5.7)	
Other race	400 (6.9)	412 (6.4)	430 (7.4)	452 (6.8)	
**Marital status (n, %)**					
Married or cohabitation	2989 (68.5)	2854 (68.8)	2748 (70.9)	2567 (64.9)	< 0.001
Widowed or separated	658 (11.6)	590 (11.3)	493 (10.0)	497 (10.8)	
Never married	971 (19.9)	924 (19.9)	842 (19.1)	1082 (24.3)	
**Education level (n, %)**					
Less than 12th grade	1395 (18.3)	1276 (16.9)	1179 (17.8)	1279 (17.0)	0.018
High school or equivalent	1194 (25.3)	1117 (25.8)	954 (22.2)	1043 (25.0)	
College graduate or above	2222 (56.3)	2148 (57.3)	2125 (60.0)	2082 (58.0)	
**Smoking status (n, %)**					
Never	2148 (49.9)	2056 (49.9)	1995 (51.5)	1925 (50.0)	0.34
Ever	1336 (27.1)	1271 (28.1)	1191 (28.5)	1166 (28.4)	
Current	1062 (23.0)	955 (22.1)	827 (20.0)	937 (21.6)	
Family PIR	3.01 ± 0.04	3.11 ± 0.04	3.26 ± 0.04	3.19 ± 0.04	< 0.001
Energy intake (kcal/day)	2384 ± 16.0	2447 ± 15.3	2425 ± 15.0	2429 ± 14.8	0.07
HEI-2015	48.45 ± 0.28	49.48 ± 0.31	49.72 ± 0.30	49.53 ± 0.30	0.004
Iron intake (mg/day)	16.64 ± 0.17	17.27 ± 0.18	16.97 ± 0.20	16.59 ± 0.17	0.52
Leisure activity (MET-min/day)	214.3 ± 6.17	240.1 ± 6.77	234.2 ± 7.18	265.8 ± 8.76	< 0.001
BMI (kg/m^2^)	29.66 ± 0.14	28.93 ± 0.12	28.35 ± 0.10	27.33 ± 0.11	< 0.001
ALT (U/L)	27.45 ± 0.32	28.94 ± 0.31	30.26 ± 0.40	30.59 ± 0.38	< 0.001
AST (U/L)	25.45 ± 0.19	26.13 ± 0.26	26.59 ± 0.23	27.43 ± 0.27	< 0.001
GGT (U/L)	29.02 ± 0.45	30.00 ± 0.51	32.01 ± 0.68	33.18 ± 0.66	< 0.001
Lead (μg/dL)	1.77 ± 0.03	1.79 ± 0.04	1.84 ± 0.04	1.92 ± 0.05	0.006
Cadmium (nmol/L)	4.07 ± 0.11	3.91 ± 0.10	3.85 ± 0.10	4.15 ± 0.11	0.65
NAFLD (n, %)	2577 (55.3)	2295 (52.2)	2062 (49.9)	1874 (44.5)	< 0.001
AHF (n, %)	237 (3.4)	179 (2.9)	131 (2.5)	99 (1.7)	< 0.001

Data were presented as survey weighted mean ± standard error for continuous variables and n (%) for categorical variables.

*AHF* advanced hepatic fibrosis, *ALT* alanine transaminase, *AST* aspartate aminotransferase, *BMI* body mass index, *GGT* gamma glutamyl transferase, *HEI* healthy eating index, *MET* metabolic equivalent of task (MET-min/day), *NAFLD* non-alcoholic fatty liver disease, *PIR* poverty income ratio.

*Unadjusted *P* values.

**Table 2:** Basic characteristics of participants according to quartiles of serum iron in females.

	Quartile 1	Quartile 2	Quartile 3	Quartile 4	P*
Serum iron (μg/dL)	< 56.0	56.0–75.5	75.6–99.5	≥ 99.6	
Age (year)	42.66 ± 0.29	47.25 ± 0.32	47.31 ± 0.33	44.43 ± 0.37	< 0.001
**Race (n, %)**					
Mexican American	1128 (11.1)	895 (7.4)	944 (7.8)	802 (6.9)	< 0.001
Other Hispanic	483 (6.9)	481 (6.6)	426 (5.9)	374 (5.2)	
Non-Hispanic White	1652 (57.6)	1984 (66.9)	2081 (70.5)	2111 (74.4)	
Non-Hispanic Black	1484 (18.1)	1140 (12.6)	838 (9.2)	517 (5.9)	
Other Race	375 (6.2)	387 (6.5)	422 (6.7)	465 (7.6)	
**Marital status (n, %)**					
Married or cohabitation	2524 (58.5)	2559 (62.2)	2452 (60.0)	2345 (62.9)	< 0.001
Widowed or separated	1214 (22.0)	1295 (22.8)	1242 (23.1)	873 (18.5)	
Never married	1138 (19.5)	845 (15.0)	848 (16.9)	856 (18.5)	
**Education level (n, %)**					
Less than 12th grade	1497 (19.8)	1262 (16.2)	1180 (15.7)	917 (12.9)	< 0.001
High school or equivalent	1218 (25.0)	1140 (24.2)	1090 (23.1)	988 (22.4)	
College graduate or above	2403 (55.2)	2478 (59.5)	2438 (61.1)	2361 (64.7)	
**Smoking status (n, %)**					
Never	3216 (65.2)	3074 (62.8)	2913 (61.3)	2544 (61.3)	0.010
Ever	732 (17.2)	882 (21.0)	813 (20.6)	742 (19.6)	
Current	799 (17.6)	720 (16.3)	734 (18.1)	708 (19.0)	
Family PIR	2.64 ± 0.04	2.94 ± 0.04	2.98 ± 0.05	3.20 ± 0.04	< 0.001
Energy intake (kcal/day)	1907 ± 12.3	1864 ± 12.6	1882 ± 11.2	1847 ± 12.6	0.005
HEI-2015	49.43 ± 0.29	51.11 ± 0.29	51.51 ± 0.33	52.2 ± 0.31	< 0.001
Iron intake (mg/day)	13.26 ± 0.13	13.29 ± 0.14	13.56 ± 0.13	13.22 ± 0.14	0.79
Leisure activity (MET-min/day)	141.0 ± 4.60	143.4 ± 4.73	167.6 ± 6.49	179.0 ± 5.63	< 0.001
BMI (kg/m^2^)	31.10 ± 0.15	29.64 ± 0.16	28.08 ± 0.14	26.19 ± 0.13	< 0.001
ALT (U/L)	20.26 ± 0.65	21.35 ± 0.27	21.33 ± 0.24	20.98 ± 0.22	0.32
AST (U/L)	21.77 ± 0.19	22.91 ± 0.24	22.98 ± 0.18	23.15 ± 0.26	< 0.001
GGT (U/L)	20.92 ± 0.38	22.14 ± 0.40	23.10 ± 0.51	23.14 ± 0.94	0.021
Lead (μg/dL)	1.17 ± 0.02	1.28 ± 0.02	1.30 ± 0.02	1.28 ± 0.02	< 0.001
Cadmium (nmol/L)	4.61 ± 0.12	4.34 ± 0.09	4.69 ± 0.10	4.71 ± 0.12	0.19
NAFLD (n, %)	2471 (47.2)	2128 (41.9)	1735 (34.4)	1130 (23.7)	< 0.001
AHF (n, %)	202 (3.0)	198 (3.1)	157 (2.4)	83 (1.6)	< 0.001

Data were presented as survey weighted mean ± standard error for continuous variables and n (%) for categorical variables.

*AHF* advanced hepatic fibrosis, *ALT* alanine transaminase, *AST* aspartate aminotransferase, *BMI* body mass index, *GGT* gamma glutamyl transferase, *HEI* healthy eating index, *MET* metabolic equivalent of task (MET-min/day), *NAFLD* non-alcoholic fatty liver disease, *PIR* poverty income ratio.

*Unadjusted *P* values.

The serum iron, ferritin, and TFR levels of participants with and without NAFLD are shown in Table 3. Participants with NAFLD had significantly lower serum iron and higher ferritin than the comparison group. TFR was also significantly higher among females with NAFLD.

**Table 3:** Iron status according to the presence of NAFLD.

	NAFLD (−)	NAFLD (+)	P*
**Males**			
Serum iron (μg/dL)	98.76 ± 0.63	91.94 ± 0.46	< 0.001
Ferritin (μg/L)	156.89 ± 4.50	213.88 ± 5.61	< 0.001
TFR (mg/L)	2.93 ± 0.08	3.10 ± 0.05	0.06
**Females**			
Serum iron (μg/dL)	85.18 ± 0.49	72.28 ± 0.48	< 0.001
Ferritin (μg/L)	58.36 ± 0.95	83.29 ± 1.91	< 0.001
TFR (mg/L)	3.39 ± 0.04	3.80 ± 0.04	< 0.001

NAFLD (−), participants without NAFLD; NAFLD (+), participants with NAFLD;

*NAFLD* non-alcohol fatty liver disease, *TFR* transferrin receptor

*Unadjusted *P* values.

The associations between serum iron, ferritin, TFR and risk of NAFLD and AHF were shown in Table 4 by a multivariable logistic regression model. After multivariable adjustment for sociodemographic factors, lifestyle behavior, diet quality, and other metal (model 3), serum iron was negatively associated with the presence of NAFLD (OR = 0.87, 95% CI 0.83–0.91, *P* < 0.001 for males; OR = 0.75, 95% CI 0.71–0.78, *P* < 0.001 for females). On the contrary, ferritin was positively associated with risk of NAFLD (OR = 1.44, 95% CI 1.30–1.61, *P* < 0.001 for males; OR = 1.36, 95% CI 1.28–1.45, *P* < 0.001 for females). Serum TFR was significantly correlated with higher risk of NAFLD only in females (OR = 1.40, 95% CI 1.30–1.50, *P* < 0.001) but not in males.

**Table 4:** Association between serum iron, ferritin, TFR (per SD increment) and the presence of NAFLD or AHF.

	Cases/participants	Model 1 OR (95% CI)	*P*	Model 2 OR (95% CI)	*P*	Model 3 OR (95% CI)	*P*
**NAFLD**							
**Males**							
Serum iron	8808/18,031	0.86 (0.82–0.89)	< 0.001	0.87 (0.83–0.91)	< 0.001	0.87 (0.83–0.91)	< 0.001
Ferritin	2456/5044	1.46 (1.32–1.62)	< 0.001	1.45 (1.30–1.62)	< 0.001	1.44 (1.30–1.61)	< 0.001
TFR	926/1675	1.22 (0.98–1.52)	0.08	1.23 (0.97–1.56)	0.08	1.22 (0.95–1.56)	0.12
**Females**							
Serum iron	7464/18,989	0.73 (0.69–0.76)	< 0.001	0.75 (0.72–0.78)	< 0.001	0.75 (0.71–0.78)	< 0.001
Ferritin	3908/11,006	1.34 (1.27–1.43)	< 0.001	1.37 (1.29–1.45)	< 0.001	1.36 (1.28–1.45)	< 0.001
TFR	2632/7419	1.38 (1.29–1.48)	< 0.001	1.37 (1.28–1.47)	< 0.001	1.40 (1.30–1.50)	< 0.001
**AHF**							
**Males**							
Serum iron	646/17,935	0.86 (0.77–0.95)	0.005	0.89 (0.79–0.99)	0.036	0.89 (0.79–1.00)	0.055
Ferritin	162/5031	1.00 (0.74–1.37)	0.99	1.00 (0.74–1.37)	0.99	0.97 (0.71–1.32)	0.84
TFR	110/1665	2.22 (1.46–3.40)	< 0.001	2.16 (1.46–3.21)	< 0.001	2.13 (1.39–3.24)	< 0.001
**Females**							
Serum iron	640/18,913	0.82 (0.74–0.92)	< 0.001	0.87 (0.78–0.98)	0.023	0.87 (0.77–0.98)	0.025
Ferritin	196/10,963	1.05 (0.79–1.39)	0.75	1.04 (0.78–1.38)	0.81	1.01 (0.75–1.36)	0.94
TFR	134/7382	1.72 (1.39–2.12)	< 0.001	1.68 (1.35–2.09)	< 0.001	1.73 (1.37–2.17)	< 0.001

Model 1: age (years), race (Mexican American, other Hispanic, non-Hispanic white, non-Hispanic black, other race), marital status (married or cohabitation, widowed or separated, never married), education level (less than 12th grade, high school or equivalent, college graduate or above), family poverty income ratio; Model 2: model 1 + smoking status (never, ever, current), alcohol intake (drinkers/week), leisure activity (MET-min/day), total energy intake (kcal/day), healthy eating index-2015; Model 3: model 2 + lead (μg/dL), cadmium (nmol/L).

*AHF* advanced hepatic fibrosis, *MET* metabolic equivalent, *NAFLD* non-alcohol fatty liver disease, *SD* standard deviation, *TFR* transferrin receptor.

After adjusting for potential confounding factors, serum iron was negatively associated with the risk of AHF only in females (OR = 0.87, 95% CI 0.77–0.98, *P* = 0.025). Serum TFR but not ferritin exhibited a significant positive association with AHF risk in males (OR = 2.13, 95% CI 1.39–3.24, *P* < 0.001) and females (OR = 1.73, 95% CI 1.37–2.17, *P* < 0.001).

Serum iron, as the main available of interest, was divided into four groups based on quartiles. As shown in Table 5, serum iron levels were significantly and inversely associated with the odds of NAFLD in males (Q4 vs. Q1: OR = 0.70, 95% CI 0.63–0.79; *P-trend* < 0.001) and in females (Q4 vs. Q1: OR = 0.39, 95% CI 0.34–0.45; *P-trend* < 0.001) after full adjustment in model 3. Serum iron levels were associated with lower odds of AHF only in females (Q4 vs. Q1: OR = 0.64, 95%, CI 0.43–0.97; *P-trend* = 0.018). Although AHF risk was not significantly decreased among Q2-Q4 participants compared with Q1, but the ORs showed a downward trend in males (*P-trend* = 0.046).

**Table 5:** Survey weighted odds ratios (95% CI) for association between serum iron and presence of NAFLD or AHF.

	Quartile for serum iron				*P*-trend
	Quartile 1	Quartile 2	Quartile 3	Quartile 4	
**NAFLD**					
**Males**					
Cases/*N*	2577/4815	2295/4545	2062/4264	1874/4407	
Model 1	1.00 (Ref.)	0.86 (0.77–0.97)	0.78 (0.70–0.88)	0.67 (0.60–0.75)	< 0.001
Model 2	1.00 (Ref.)	0.89 (0.79–0.99)	0.80 (0.71–0.90)	0.69 (0.62–0.78)	< 0.001
Model 3	1.00 (Ref.)	0.89 (0.79–1.00)	0.81 (0.72–0.91)	0.70 (0.63–0.79)	< 0.001
**Females**					
Cases/*N*	2471/5122	2128/4887	1735/4711	1130/4269	
Model 1	1.00 (Ref.)	0.77 (0.69–0.86)	0.55 (0.50–0.62)	0.36 (0.32–0.41)	< 0.001
Model 2	1.00 (Ref.)	0.78 (0.69–0.87)	0.58 (0.51–0.65)	0.39 (0.34–0.45)	< 0.001
Model 3	1.00 (Ref.)	0.78 (0.69–0.87)	0.58 (0.51–0.65)	0.39 (0.34–0.45)	< 0.001
**AHF**					
**Males**					
Cases/*N*	237/4796	179/4522	131/4235	99/4382	
Model 1	1.00 (Ref.)	0.88 (0.64–1.20)	0.76 (0.55–1.06)	0.63 (0.44–0.90)	0.006
Model 2	1.00 (Ref.)	0.92 (0.67–1.26)	0.80 (0.57–1.12)	0.70 (0.49–1.01)	0.031
Model 3	1.00 (Ref.)	0.92 (0.66–1.26)	0.79 (0.56–1.12)	0.72 (0.50–1.04)	0.046
**Females**					
Cases/*N*	202/5102	198/4869	157/4690	83/4252	
Model 1	1.00 (Ref.)	0.80 (0.58–1.10)	0.64 (0.46–0.89)	0.52 (0.35–0.79)	< 0.001
Model 2	1.00 (Ref.)	0.82 (0.59–1.13)	0.70 (0.50–0.98)	0.64 (0.42–0.96)	0.015
Model 3	1.00 (Ref.)	0.81 (0.59–1.12)	0.70 (0.50–0.98)	0.64 (0.43–0.97)	0.018

Model 1: age (years), race (Mexican American, other Hispanic, non-Hispanic white, non-Hispanic black, other race), marital status (married or cohabitation, widowed or separated, never married), education level (less than 12th grade, high school or equivalent, college graduate or above), family poverty income ratio; Model 2: model 1 + smoking status (never, ever, current), alcohol intake (drinkers/week), leisure activity (MET-min/day), total energy intake (kcal/day), healthy eating index-2015; Model 3: model 2 + lead (μg/dL), cadmium (nmol/L).

*AHF* advanced hepatic fibrosis, *MET* metabolic equivalent, *NAFLD* non-alcohol fatty liver disease.

**Figure 1:**
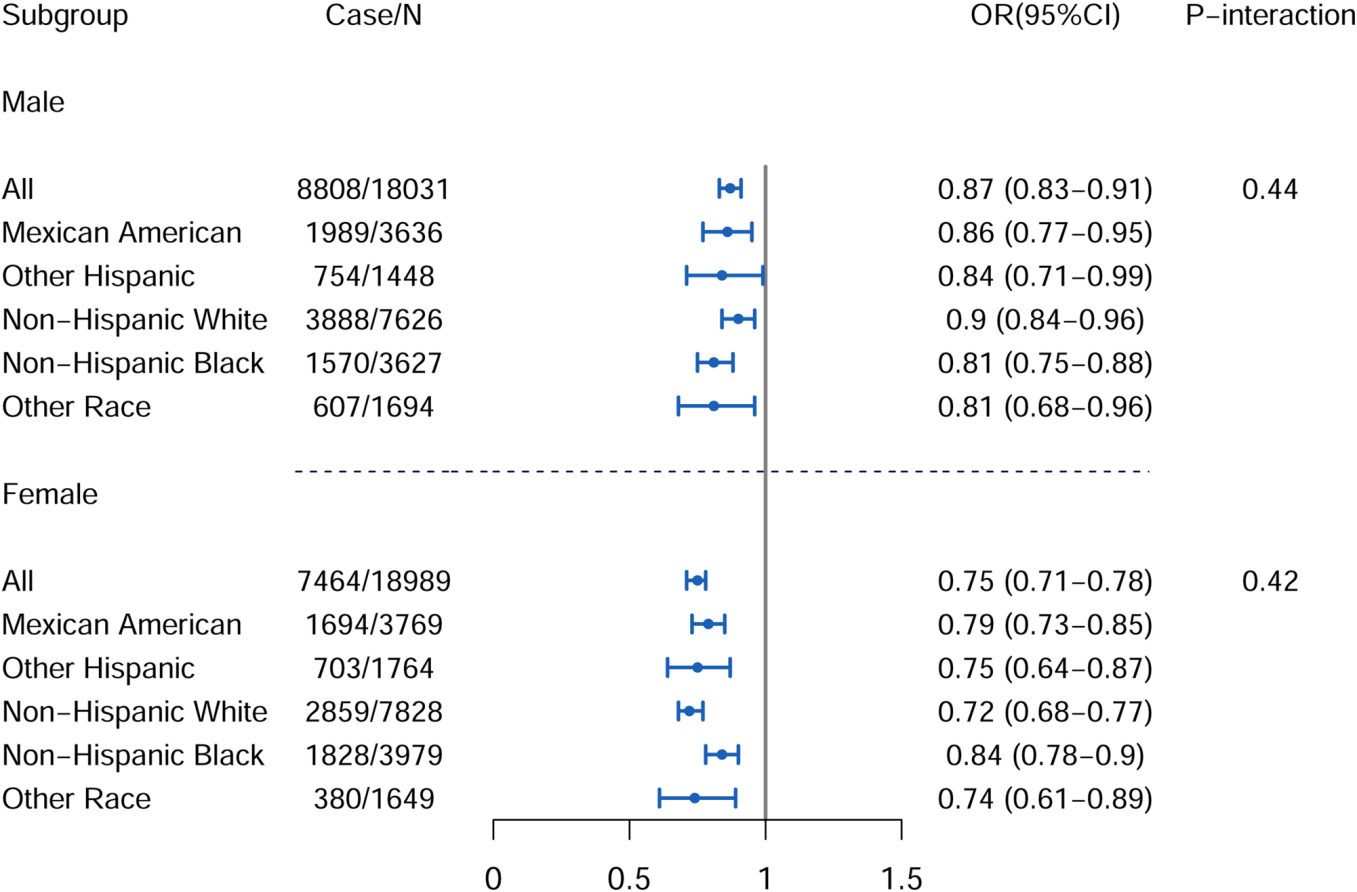
Race-stratified analysis of association between serum iron, with per SD increase, and presence of NAFLD. Adjusted for Model 3: age (years), race (Mexican American, other Hispanic, non-Hispanic white, non-Hispanic black, other race), marital status (married or cohabitation, widowed or separated, never married), education level (less than 12th grade, high school or equivalent, college graduate or above), family poverty income ratio, smoking status (never, ever, current), alcohol intake (drinkers/week), leisure activity (MET-min/day), total energy intake (kcal/day), healthy eating index-2015, lead (μg/dL), cadmium (nmol/L); *MET* metabolic equivalent, *SD* standard deviation, *NAFLD* nonalcoholic fatty liver disease. The figure was created by RStudio (Version 1.3.1056, URL: https://www.rstudio.com/).

**Figure 2:**
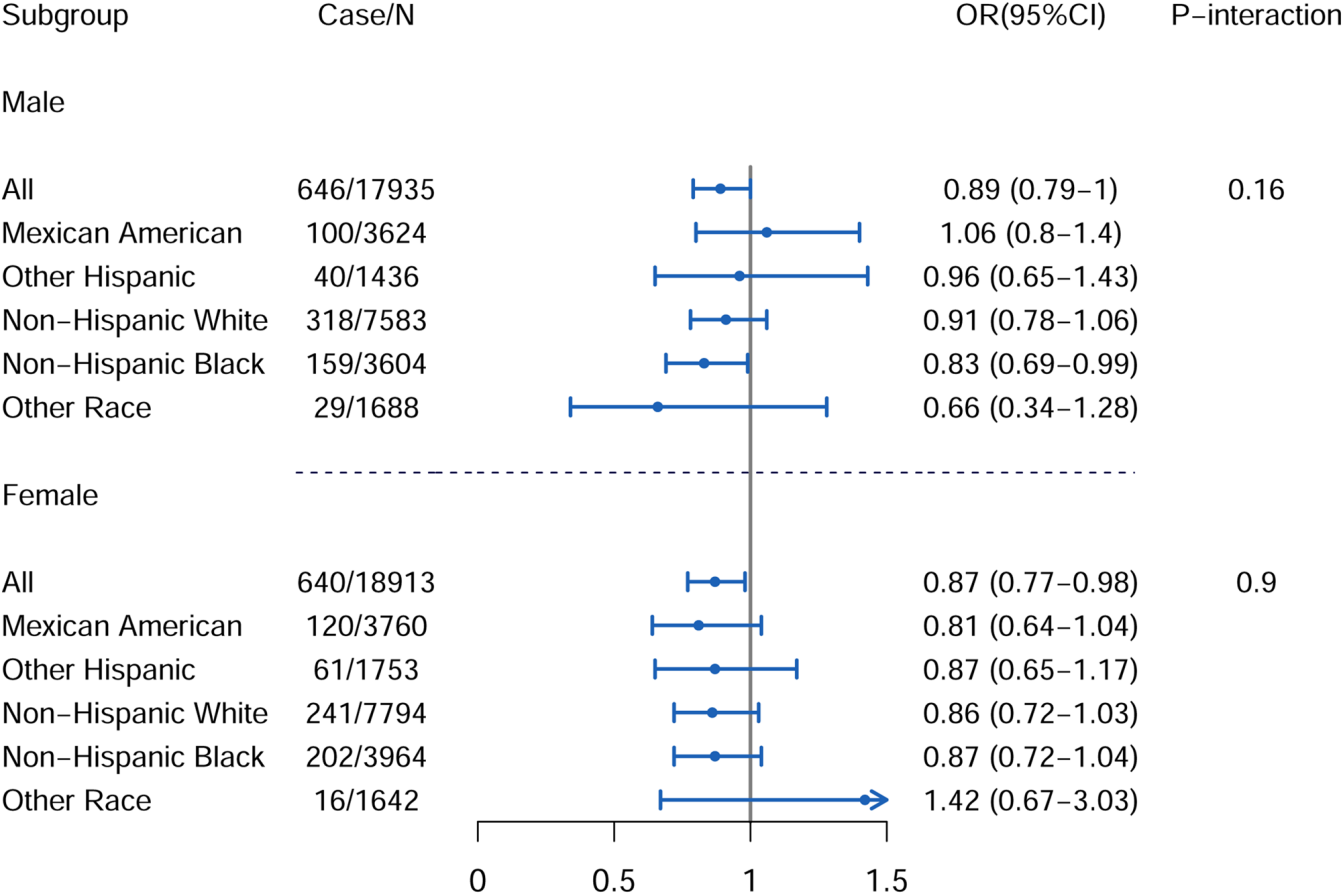
Race-stratified analysis of association between serum iron, with per SD increase, and presence of AHF. Adjusted for model 3: age (years), race (Mexican American, other Hispanic, non-Hispanic white, non-Hispanic black, other race), marital status (married or cohabitation, widowed or separated, never married), education level (less than 12th grade, high school or equivalent, college graduate or above), family poverty income ratio, smoking status (never, ever, current), alcohol intake (drinkers/week), leisure activity (MET-min/day), total energy intake (kcal/day), healthy eating index-2015, lead (μg/dL), cadmium (nmol/L); *AHF* advanced hepatic fibrosis, *MET* metabolic equivalent, *SD* standard deviation. The figure was created by RStudio (Version 1.3.1056, URL: https://www.rstudio.com/).

We conducted a subgroup analysis by race/ethnicity of the study population. As shown in Fig. 1 (for NAFLD) and Fig. 2 (for AHF), the associations between serum iron and risk of NAFLD and AHF, though under different magnitudes, were broadly consistent across different racial/ethnical groups, and no significant interactions were observed (*P-interaction* > 0.10).

## Discussion

In a nationally representative sample of US adults, our study assessed the cross-sectional relationships between serum iron status indicators and risk of liver diseases. Our findings suggested that higher level of serum iron was associated with lower risk of NAFLD in both genders and lower risk of AHF only in females after multivariable adjustment. No significant racial/ethnical difference in these associations was observed.

Findings relating to the relationship between serum ferritin, TFR and the risk of liver injury have been reported^9–12^. We also found the positive association between serum ferritin, TFR and liver diseases risk. Serum ferritin was significantly correlated with higher risk of NAFLD but not of AHF, and serum TFR was positively related to AHF in both genders and to NAFLD in females.

As main results, the inverse relationship between serum iron and the presence of NAFLD should be highlighted in the present study. Consistent with our results, previous epidemiological studies demonstrated that serum iron was inversely associated with risk of cardiovascular disease^23^, chronic alcoholic liver disease^6^ and diabetic retinopathy^24^. The pathogenic mechanisms underlying the association are not well understood. Serum iron is highly dynamic in vivo, and the labile iron pool amounts to 4 mg iron, less than 0.2% of total iron^25^. The body would store iron in ferritin form when serum iron is too high or iron requirement is reduced^26^. Conversely, iron stores are depleted and the body is in a severe iron deficiency state if there is a reduction in serum iron concentration^27^. Iron plays an important role for mitochondrial respiratory chain complexes, and iron deficiency lead to an overall impairment of mitochondrial respiration, which is important for fatty acid metabolism. It is well known that intrahepatic lipid accumulation is one of the main characterizations of NAFLD^28^. Thus, the lack of iron results in higher susceptibility for lipid accumulation in hepatocyte. This may be one of the major reasons for the negative association between serum iron and liver diseases.

In addition, several other plausible explanations may account for the inverse association between serum iron and NAFLD. First, hepcidin is the master regulator to maintain iron homeostasis; it is responsible for iron transport from the intracellular space into systematic circulation^29^. Its expression decreases when the liver is injured. Thus, the process of iron release from cells to plasma is blocked^30^. Furthermore, hepcidin is up-regulated by interleukin-6 in acute and chronic inflammation, leading to hypoferremia^31–33^. Therefore, lower serum iron was associated with chronic liver disease, such as NAFLD.

About one third of NAFLD patients are accompanied by iron overload, called dysmetabolic iron overload syndrome (DIOS), which is characterized by elevated ferritin^34^. Several epidemiological studies have confirmed this conclusion. Mörwald *et.al* reported that liver fat was positively correlated with serum ferritin, but not associated with serum transferrin or iron in male adolescents with obesity^12^; Sabrina *et.al* revealed that the serum iron:ferritin ratio was associated with reduced risk of severe fatty liver progression in young adult women^35^. Thus, higher levels of serum iron may be related to lower storage of iron in a ferritin form to some extent. In the present study, the risk of NAFLD was inversely associated with serum iron and positively with ferritin concentrations, which was consistent with the above researches.

The major highlights of this study are the relatively large and well-designed population-based sample, and the nationally representative nature of the participants increases the generalizability of the findings. Our findings suggest that a more specific focus on the serum iron level for chronic liver disease patients is required and vice versa.

However, several limitations are as follows. First and foremost, the diagnosis indexes (i.e., the FLI and NFS) used in our study are not the gold standards for the diagnosis of NAFLD and AHF. Patients with hereditary disorders such as Wilson’s disease, Reye’s syndrome should be excluded according to the definition of NAFLD^16^, but information on those disorders is not available in NHANES. Furthermore, NFS is a better tool to exclude than identify AHF owing to its high negative predictive values^36^. Hence, the prevalence of AHF is likely underestimated. In addition, it has been reported the low diagnostic accuracy of NFS for those aged ≤ 35 or ≥ 65 years, lean, and morbidly obese patients^37,38^. Coupled with the relatively small of AHF cases, our results for the association between serum iron and AHF should be interpreted with caution. Secondly, all data were acquired in a time span of 20 years (1999–2018), and whether this large time span may have introduced some biases is unclear. Third, as an observational study, potential influence of residual confounding on our findings cannot be completely excluded. And we cannot identify the temporal relationship between serum iron and NAFLD because of the cross-sectional design of the analysis. In other words, reduced serum iron may be a consequence rather than a cause of liver diseases that we assessed. Thus, further studies such as large prospective cohort studies and Mendelian randomization analyses, are still needed to confirm these findings and to clarify the causal relationship.

## Conclusions

In summary, our findings suggested that higher serum iron levels were associated with lower risk of NAFLD in males and females, and with lower risk of AHF in females but not in males. No significant racial/ethnical differences in these associations were observed.

## Supplementary Information

Supplementary Information 1.

Supplementary Information 2.

## Data Availability

The datasets generated during and/or analyzed during the current study are available in the National Health and Nutrition Examination Survey repository, [https://www.cdc.gov/nchs/nhanes/index.htm].
