# Supplementary material for: Serum iron and risk of nonalcoholic fatty liver disease and advanced hepatic fibrosis in US adults
Source: Sci Rep. 2021 May 17;11:10387. doi: 10.1038/s41598-021-89991-x (PMC8128903; doi:10.1038/s41598-021-89991-x)

Serum iron and risk of nonalcoholic fatty liver disease and advanced hepatic fibrosis in US adults

Huan-Huan Yang [1], Guo-Chong Chen [2], De-Ming Li [1], Lei Lan [3], Li-Hua Chen [4], Jia-Ying Xu [5], Li-Qiang Qin *[,1]

Supplementary Figure S1. Participants Flow Chart.

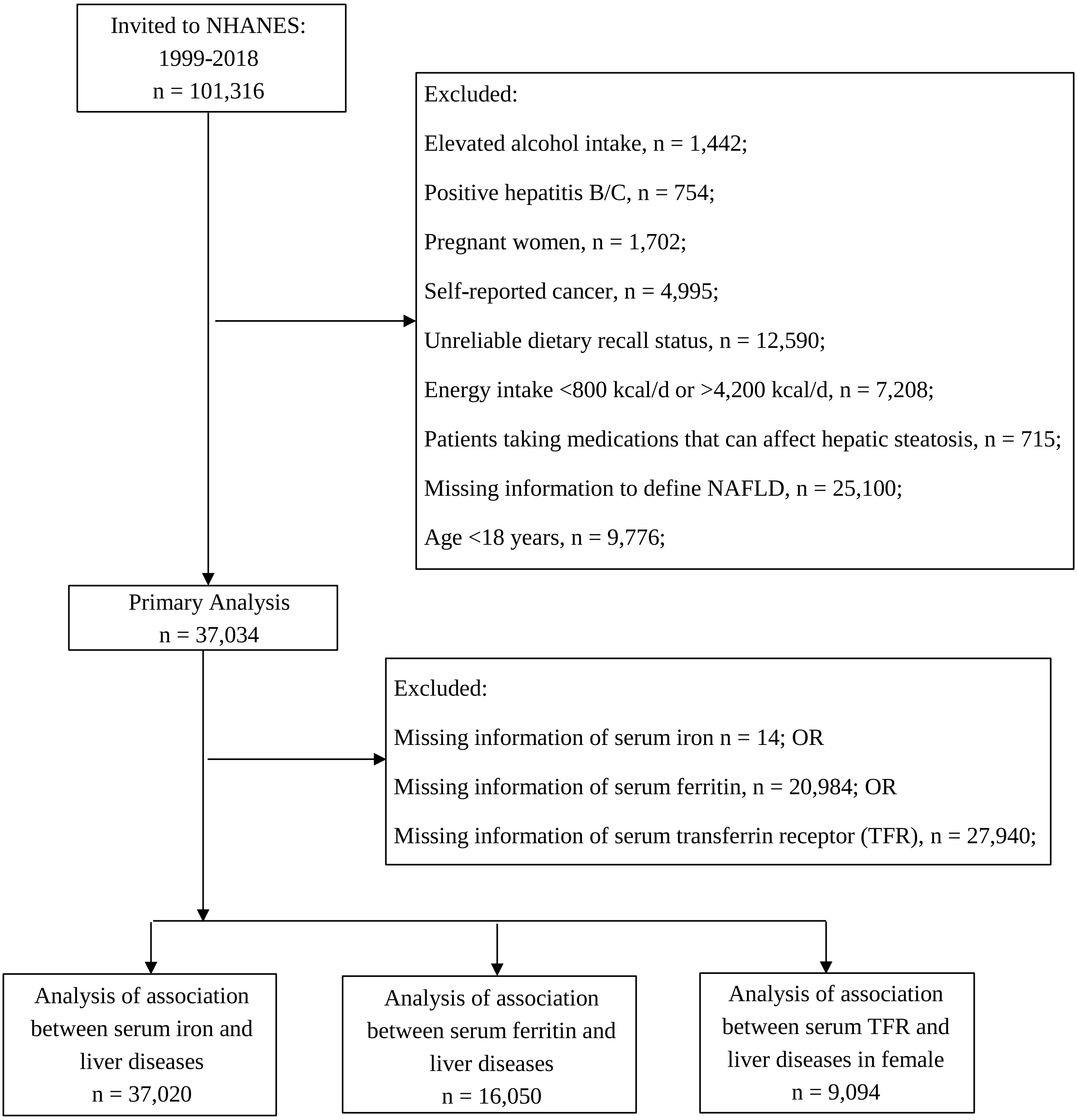

Supplement: Supplementary file 1 — Supplementary Information 1. [file 41598_2021_89991_MOESM1_ESM.pdf]
