# Supplementary material for: Serum iron and risk of nonalcoholic fatty liver disease and advanced hepatic fibrosis in US adults
Source: Sci Rep. 2021 May 17;11:10387. doi: 10.1038/s41598-021-89991-x (PMC8128903; doi:10.1038/s41598-021-89991-x)
Supplement: Supplementary file 2 — Supplementary Information 2. [file 41598_2021_89991_MOESM2_ESM.pdf]

Serum iron and risk of nonalcoholic fatty liver disease and advanced hepatic fibrosis in US adults

Huan-Huan Yang [1], Guo-Chong Chen [2], De-Ming Li [1], Lei Lan [3], Li-Hua Chen [4], Jia-Ying Xu [5], Li-Qiang Qin *,[1]

Supplementary Table S1. Healthy Eating Index (HEI)-2015 components, point values, and standards for scoring.

**Component [**14]	**Standard for minimum score of zero**	**Standard for maximum score**	**Maximum points**
Total fruits	0	≥ 0.8 cup equivalent	5
Whole fruits	0	≥ 0.4 cup equivalent	5
Total vegetables	0	≥ 1.1 cup equivalent	5
Greens and beans	0	≥ 0.2 cup equivalent	5
Whole grains	0	≥ 1.5 cup equivalent	10
Total dairy	0	≥ 1.3 cup equivalent	10
Total protein foods	0	≥ 2.5 oz equivalent	5
Seafood and plant proteins	0	≥ 0.8 oz equivalent	5
Fatty acids	(PUFA + MUFA) / SFA ≤ 1.2	(PUFA + MUFA) / SFA ≥ 2.5	10
Refined grains	≥ 4.3 oz equivalent	≤ 1.8 oz equivalent	10
Sodium	≥ 2.0 g	≤ 1.1 g	10
Added sugar	≥ 26% of energy	≤ 6.5% of energy	10
Saturated fats	≥ 16% of energy	≤ 8% of energy	10
Total scores	0		100

MUFA, monounsaturated fatty acids; PUFA, polyunsaturated fatty acids; SFA, saturated fatty acids; Each of the food groups was corrected for dietary energy 1,000 kcal.
